# Kidney transplantation from living donor with monolateral renal artery fibromuscular dysplasia using a cryopreserved iliac graft for arterial reconstruction: a case report and review of the literature

**DOI:** 10.1186/s12882-020-02097-w

**Published:** 2020-10-28

**Authors:** Rostand Emmanuel Nguefouet Momo, Paola Donato, Gabriele Ugolini, Francesco Nacchia, Luca Mezzetto, Gian Franco Veraldi, Stefano Marletta, Enrico Cavallo, Albino Eccher, Annamaria Giambanco, Daniela Cenzi, Luigino Boschiero

**Affiliations:** 1 Unità Dipartimentale Trapianti di Rene, Dipartimento di Chirurgia ed Odontoiatria, Azienda Ospedaliera Universitaria Integrata di Verona, Verona, Italy; 2 Unità Operativa di Chirurgia Vascolare, Azienda Ospedaliera Universitaria Integrata di Verona, Verona, Italy; 3 Unità Operativa di Anatomia Patologica, Azienda Ospedaliera Universitaria Integrata di Verona, Verona, Italy; 4 Unita operativa di Chirugia Pediatrica, Azienda Ospedaliera Universitaria Integrata di Verona, Verona, Italy; 5 Unità Operativa di Radiologia, Dipartimento di Radiologia, Azienda Ospedaliera Universitaria Integrata di Verona, Verona, Italy

**Keywords:** Fibromuscular dysplasia, Living donor, Renal transplant, Arterial reconstruction, Cryopreserved cadaveric iliac graft

## Abstract

**Background:**

Aging and mortality of patients on waiting lists for kidney transplantation have increased, as a result of the shortage of organs available all over the world. Living donor grafts represent a significant source to maintain the donor pool, and resorting successfully to allografts with arterial disease has become a necessity. The incidence of renal artery fibromuscular dysplasia (FMD) in potential living renal donors is reported to be 2–6%, and up to 4% of them present concurrent extra-renal involvement.

**Case presentation:**

We present a case of renal transplantation using a kidney from a living donor with monolateral FMD. Resection of the affected arterial segment and its subsequent replacement with a cryopreserved iliac artery graft from a deceased donor were performed. No intraoperative nor post-operative complications were reported. The allograft function promptly resumed, with satisfying creatinine clearance, and adequate patency of the vascular anastomoses was detected by Doppler ultrasounds.

**Conclusion:**

Literature lacks clear guidelines on the eligibility of potential living renal donors with asymptomatic FMD. Preliminary assessment of the FMD living donor should always rule out any extra-renal involvement. Whenever possible, resection and reconstruction of the affected arterial segment should be taken into consideration as this condition may progress after implantation.

## Background

The shortage of organs available for renal transplantation has become a serious concern all over the world [1]. Aging and mortality of patients on waiting lists for kidney transplantation have increased [2]. In order to overcome this problem, more extended criteria donor have been used successfully with excellent graft survival and renal function recovery [3]. Living donor grafts represent a significant source to maintain the donor pool, and resorting successfully to allografts with arterial disease has become a necessity [4]. In literature, the debate about kidney transplantation from living renal donor presenting with fibromuscular dysplasia (FMD) is controversial, as non-transplantable kidneys with FMD have been reported [5]. Kidneys with FMD are generally considered to be not suitable for transplantation, because this condition may progress after implantation; FMD is the second most common pathologic finding during donor evaluation [6]. Kidney donation may be associated with a risk of long-term complications, including the onset or rapid progression of FMD in the native kidney, emphasizing the need of long term follow-up for the donor [7–9]. FMD is a fibrous non-atherosclerotic and non-inflammatory disease of medium and small caliber arteries [10]. It may be clinically silent [11] or it may cause vascular dilatation or stenosis, leading to arterial hypertension, aneurysms and thrombosis, if left untreated [11, 12]. The etiology of FMD is not well known. It is estimated that the incidence in general population is 2–3% [13]; moreover, it is reported to be 2–6% in potential living renal donors, who may present extra-renal involvement in 4% of cases [14–17]. It is also recognized that incidence is higher in women and the most common clinical presentation is hypertension. Three subtypes of FMD are described: intimal, medial and adventitial. The medial form represents the most common one. Severity of FMD is assessed on the base of the degree of the arterial stenosis. Mild FMD is defined as mild irregularity of the artery without significant stenosis, and moderate FMD is defined as arterial irregularity and stenosis less than 50% of the arterial caliber [18]. We report a case of renal transplantation using the affected kidney of a living donor with monolateral renal FMD; a resection of the pathological arterial segment and its subsequent reconstruction with a cryopreserved iliac artery graft from deceased donor were performed.

## Case presentation

**Fig. 1:**
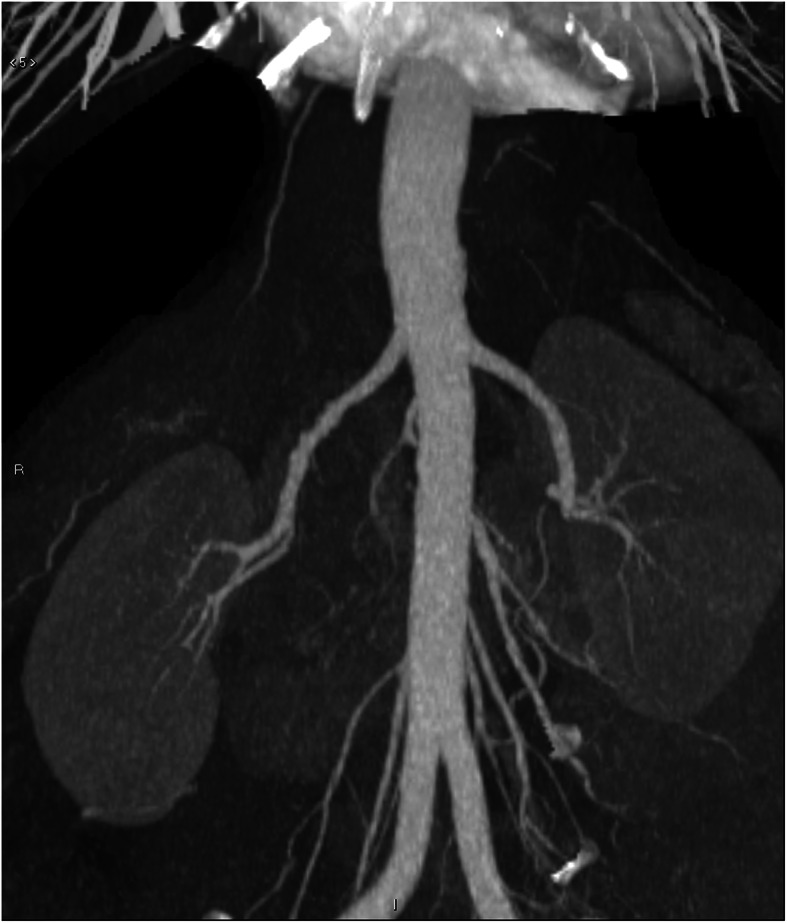
Coronal renal angiography of the living donor, showing the “string of beads” appeareance of the right renal artery

**Fig. 2:**
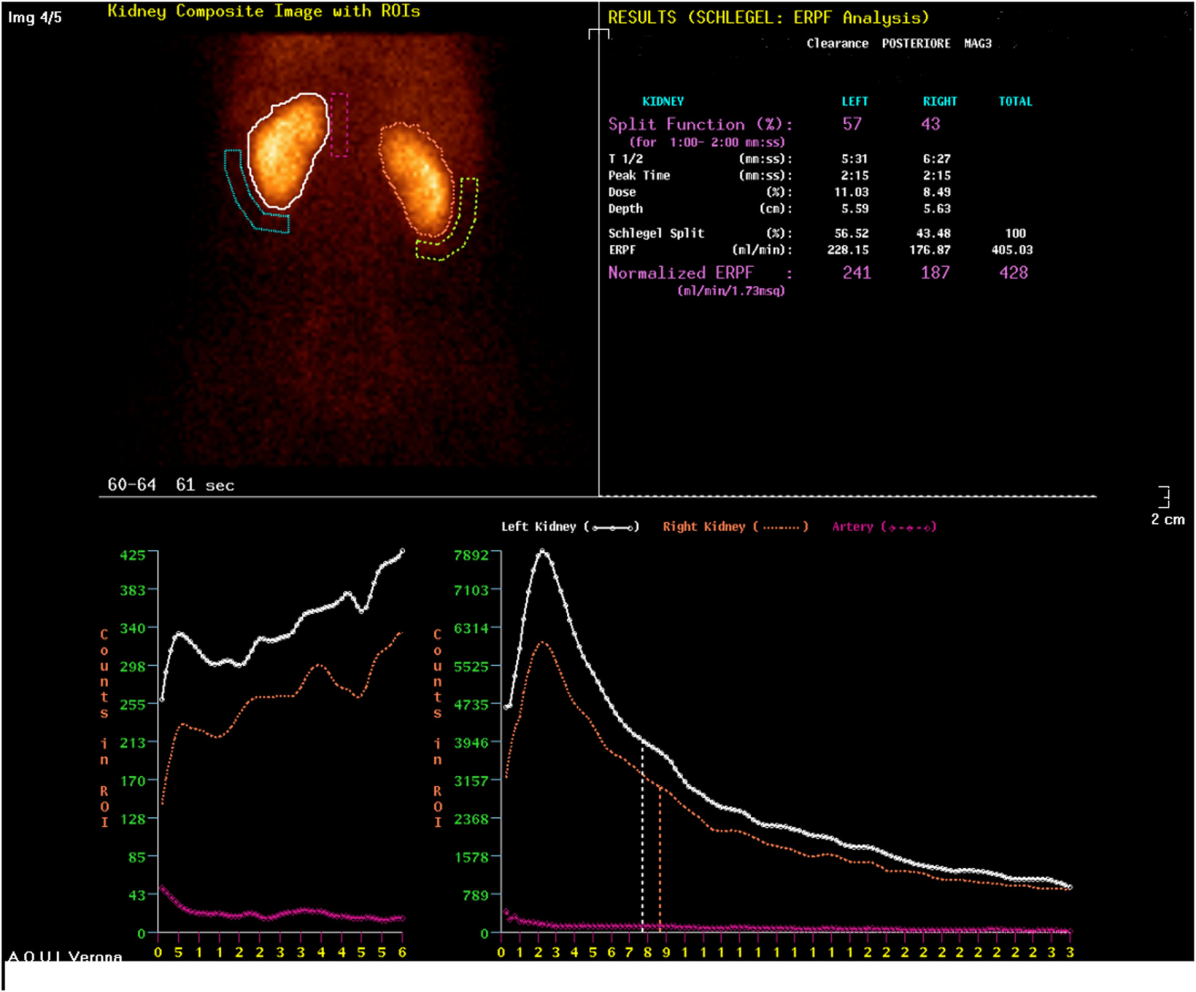
Evaluation of split renal function of the living kidney donor using conventional posterior Tc99m-mercaptoacetylgtriglycin (MAG3) scintigraphy. Right Kidney: 43%. Left Kidney: 57%

**Fig. 3:**
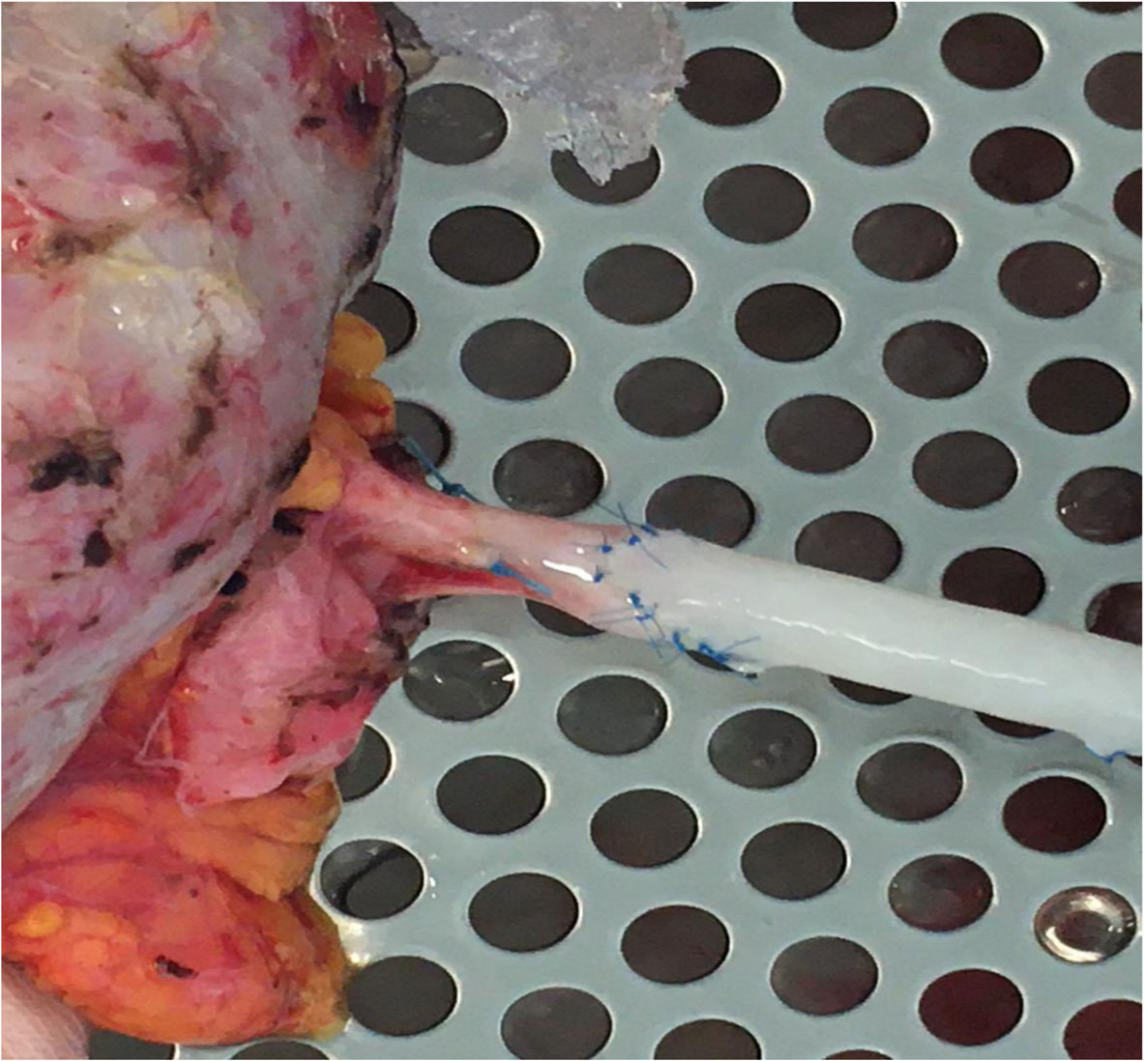
End-to-end anastomosis between the cryopreserved iliac artery graft from a deceased donor and the right renal residual safe artery of the living donor

**Fig. 4:**
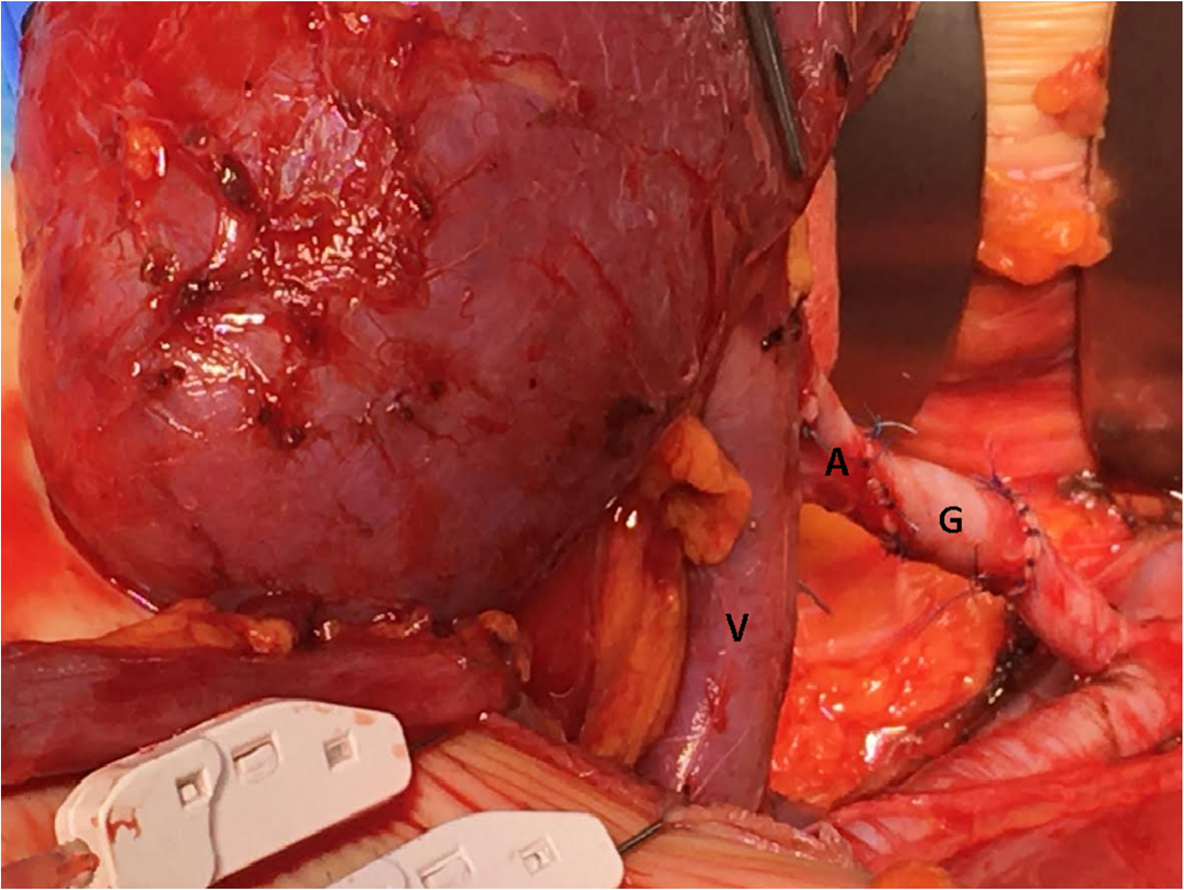
Right grafted kidney vein **(V)** and artery **(A)** with the interposition of the cryopreserved iliac artery graft from a deceased donor **(G)**, were anastomosed in situ to the iliac external vein an iliac internal artery respectively

A 25 years old Caucasian male patient with trisomy 21 and end stage renal disease, due to chronic pyelonephritis was considered for a pre-emptive kidney transplant. His creatinine level was 6.5 mg/dL; blood urea nitrogen and GFR were 95 mg/dL, and 14 ml/min respectively. The 54 years old adoptive mother was the only available donor. She had no story of hypertension nor diabetes mellitus. Her creatinine level was 0.9 mg/dL; blood urea nitrogen and GFR were 25 mg/dL and 99 ml/min respectively. Preliminary assessment for donation showed no contraindications, except for the computed tomography angiography (CTA) that revealed a single right renal artery with focal irregularity, without significant stenosis, suggesting mild monolateral arterial FMD (Fig. 1). The right kidney’s relative function assessed by Tc99m-mercaptoacetylgtriglycin scintigraphy, was 43% (Fig. 2); so it was considered by mutual agreement for retrieval. Then, any extra-renal FMD involvement was ruled out by thorough radiological assessment, including thoracic, neck, cerebral magnetic resonance imaging and ultrasonography of the supra-aortic trunks. Therefore, donor right side nephrectomy was performed. During the stage of bench surgery reconstruction, the affected 2.5 cm long segment of the renal artery was removed and sent for histological examination. The sample obtained was fixed in 10% formalin and wholly embedded in paraffin. Paraffin-embedded tissue block was cut into 2- to 3-μm sections and stained using hematoxylin–eosin and Masson’s trichrome. The residual renal artery length was considered too short and unsafe for the anastomosis. Therefore, a cryopreserved iliac artery graft from a deceased donor was used for the right renal artery end-to-end extension (Fig. 3). The cadaveric iliac graft was ABO-compatible. Since the risk of immune response was very low, no cross-matching test was performed before implantation. The kidney was transplanted to the recipient’s left iliac fossa. The renal vein was anastomosed to the external iliac vein. A cryopreserved cadaveric iliac artery graft was prepared to bridge the gap created by removing the affected segment of the donor’s renal artery. End-to-end anastomoses were performed between the vascular graft and the end of the renal artery (bench procedure) distally, and the end of the internal iliac artery of the recipient proximally (Fig. 4). Lich-Gregoir ureteral implantation technique was used to anastomose the ureter to the bladder. Interestingly, the first warm ischemia time was 3 min. The cold ischemia time, including 40 min of bench surgery stage, was 2 h and 45 min. The anastomosis time lasted 39 min. Induction immunosuppression consisted of methylprednisolone 125 mg and two doses of basiliximab 20 mg given on operative days zero and fourth. At the time of reperfusion 10 mg/kg of metilprednisolone were administered. Maintenance immunosuppression was based on triple regimen (tacrolimus, mycophenolate mofetil and prednisone). The vessel graft was not soaked in antibiotic solution before implantation, as the patient received preoperative antibiotic prophylaxis (Piperacilline).

**Fig. 5:**
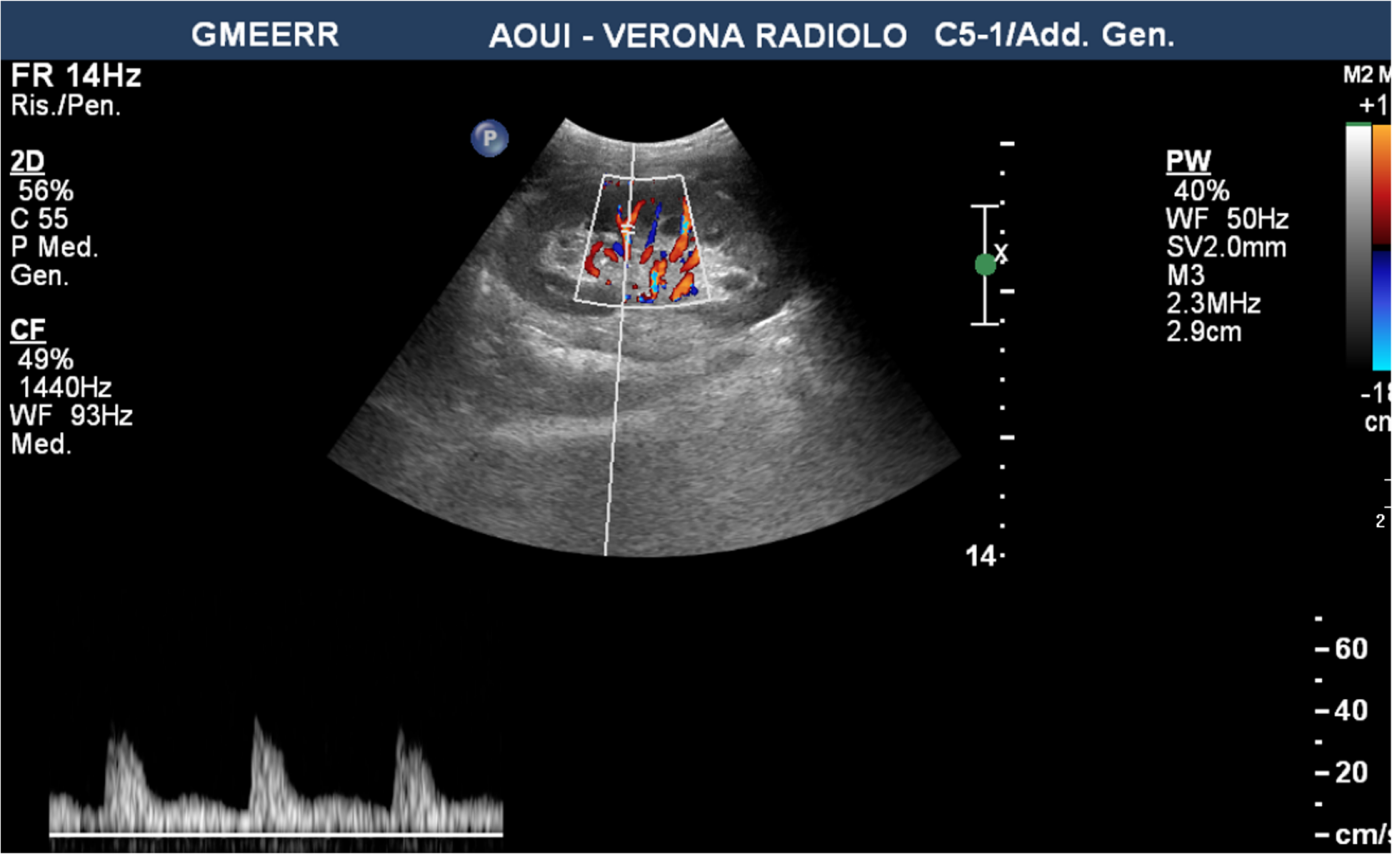
Doppler Ultrasound performed on third postoperative day demonstrating good patency of the vascular anastomoses

**Fig. 6:**
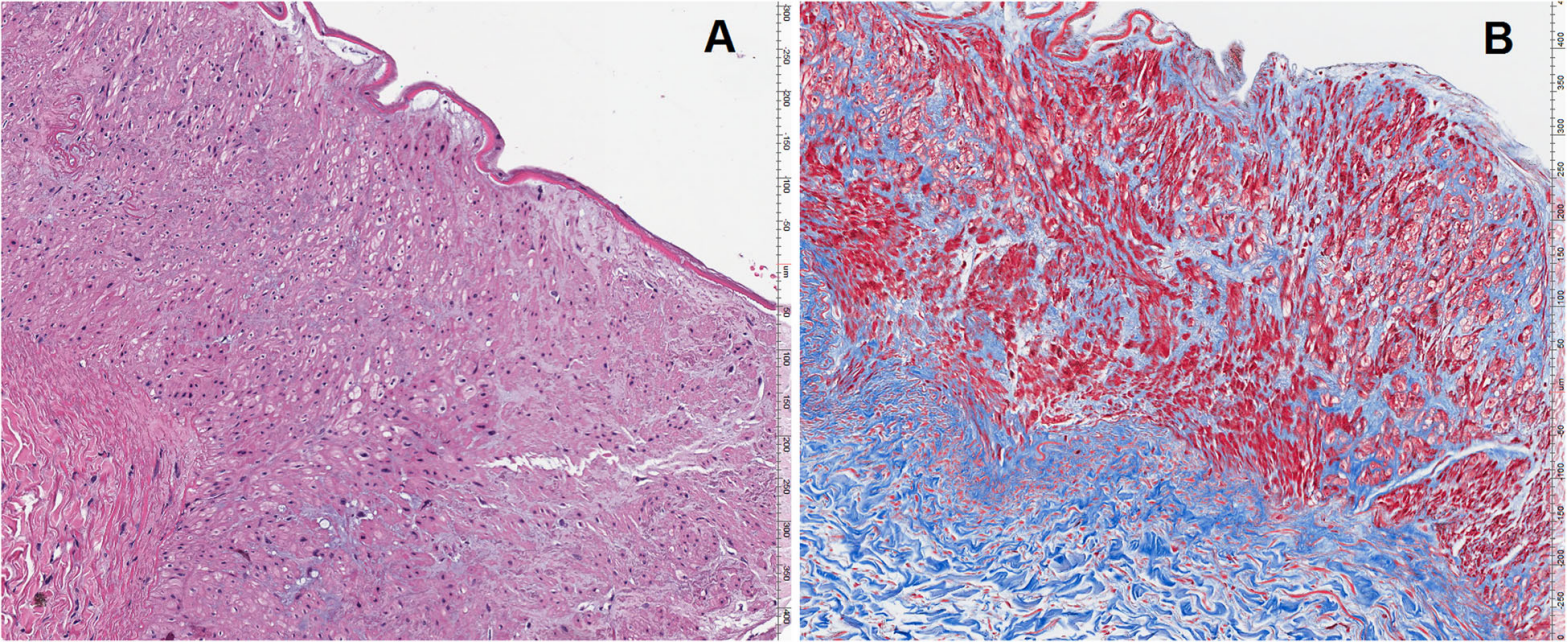
Hematoxylin and eosin staining showing proliferating smooth muscle cells haphazardly arranged within the surrounding fibrous stroma **a**, highlighted by Masson’s trichrome staining **b**, resulting in alternating thinned and thickened areas of the media of the artery wall (medium power examination, 10x)

No intraoperative nor post-operative complications were reported. The allograft function promptly resumed. In fact, on the seventh post operative day, serum creatinine level decreased from 6,5 mg/dL to 1,5 mg/dL, while glomerular filtration rate and blood urea nitrogen were 68 ml/24 Hrs and 40 mg/dL respectively. Doppler ultrasounds performed at the end of the procedure and on the first three post-operative days showed adequate patency of vascular anastomosis. (Fig. 5) The histological analysis with hematoxylin–eosin stain of the biopsy made at the time of bench surgery showed a segment of arterial wall characterized by alternating thinned and thickened areas within the media, with a haphazard arrangement and proliferation of both smooth muscle cells and the surrounding collagenous stroma, which was pointed out by the Masson’s trichrome staining. These morphological and histochemical findings were consistent with the diagnosis of FMD of the renal artery, medial fibroplasia subtype. (Fig. 6) The donor was discharged from the hospital on the fifth postoperative day with good blood pressure, serum creatinine level of 0,96 mg/dL, glomerular filtration rate of 75 ml/ 24 Hrs, and blood urea nitrogen of 29 mg/dl. After transplant, creatinine level, glomerular filtration rate and blood urea nitrogen were adequate during 9-months’ follow up of donor and recipient.

## Discussion and conclusions

We report a transplantation case of the affected kidney from a viable donor with monolateral FMD, performed by resecting and subsequently reconstructing the pathological arterial segment with a cryopreserved cadaveric iliac graft without any complication. The etiology of FMD is not completely known. It is estimated that the incidence in general population is 2–3% [13], and it is higher in women. The most common symptom is hypertension, that is more associated with bilateral renal artery FMD [17], but clinical presentation of FMD varies widely from an asymptomatic condition to a multi-system disease depending on the anatomic distribution, extent of vascular involvement and type of FMD. For this reason, on one hand, despite of thorough preoperative assessments, failure to recognize beforehand grafts with FMD occasionally occurs when it comes to living donor kidney transplantation, especially if donors are clinically asymptomatic [1]. On the other hand, considering the shortage of organs, patients with FMD should not be categorically rejected for donation but instead the decision to resort to them must be taken only after an accurate evaluation of standard criteria, such as age, comorbidities, glomerular filtration rate and creatinine level and after excluding any extra-renal involvement or surgically non correctable moderate or severe renal artery FMD [19, 20]. Our donor had no story of hypertension, and she was completely asymptomatic. As regard to donor safety, a subclinical renovascular disease like FMD has to be accurately diagnosed as well. As part of our transplant protocol, preliminary assessment for donation always includes an abdominal CTA. Due to a higher spatial resolution, CTA reaches better sensitivity than magnetic resonance angiography (97–100% versus 90–97%) [21–23]. In this case report, a diagnosis of monolateral medial FMD subtype was suspected on CTA, which showed “string of beads” appearance, defined as alternating segments of concomitant stenosis and post stenotic dilatation, involving only the right renal artery. Also, the morphological and histochemical findings were later consistent with the diagnosis of medial FMD subtype of the renal artery. We believe that the resection of the affected segment prevents long term vascular complications such as renal artery stenosis (RAS). Then, we decided to use a cryopreserved iliac artery graft from a deceased donor for the right renal artery end-to-end extension. Tondolo and colleagues [5] reported what they described as a non-transplantable cadaveric kidney with FMD, due to the unsafe correction of the intrahilar extension of multiple aneurysms. In contrast, Hyo-Sin et al. [1] reported a case of successful kidney transplantation using a deceased donor graft with severe FMD showing multiple saccular types of aneurysms and focal stenosis in the proximal renal artery, after a safe surgical correction, without resorting to a cryopreserved vascular graft. We believe that, if the renal artery left after correction is not sufficiently long for a safe anastomosis, an unaffected vessel graft could be harvested from the same cadaveric donor. In our opinion, Tondolo et al. reported a severe case of FMD, involving the hilum, which was unsafe to correct. In living donor setting such a situation is not tolerable and must be diagnosed during the preliminary assessment of the donors. In our case the renal artery was mildly involved and the segment affected was safe to be surgically corrected. Literature lacks clear guidelines on the eligibility of potential living renal donors with asymptomatic FMD. Rapid progression of mild FMD one year after transplantation have been reported, suggesting FMD cannot be considered benign in a potential normotensive renal donor [9]. Nevertheless, it has also been reported that carefully selected patients with FMD have been successfully used as renal donors [18–20, 24, 25]. In fact, among asymptomatic renal donors with FMD presenting mild irregularity and no significant stenosis of the renal artery, none of them exhibited hypertension, proteinuria, or significant changes in serum creatinine level throughout a mean follow up of 4,5 years after donation [19]. Sun et al. [18] reported a case of renal transplant from living donor using the kidney with FMD that had a higher GFR than the unaffected one, pointing out the donor safety as regard to FMD progression. Nonetheless, the Authors did not describe whether, after nephrectomy, the remaining renal artery was still affected or not, since no surgical resection and subsequent reconstruction were reported. As regard to recipients, this may be judged unsafe, since some studies assessed the natural evolution of FMD, showing worsening stenosis in 33% of cases but no case of complete arterial obstruction. However, methodological problems have been found in those studies, with a risk of progression that may have been overestimated [11, 12]. Kidneys with FMD are generally considered a contraindication for transplantation, because this condition may progress after implantation, leading to the graft loss [6]. This might be the reason why some Authors prefer to resect or repair the affected renal artery [1, 20, 24–26]. Even in a non transplant setting, surgical angioplasty is considered the ideal treatment of FMD because removal of the affected artery is only possible by surgical resection [27]. However, interventional angioplasty is preferred over surgical angioplasty because the last one is associated with high morbidity and mortality (6.3% vs 15.4 and 0.9% vs 1.2% respectively) [28, 29]. More recently, Matsushita Y. et al. reported a case on arterial grafting of the donor renal artery affected by FMD [26]; a limitation of both surgical procedures performed in their and our case was the interruption of the internal iliac artery. The use of internal iliac artery in our patient was possible as the opposite side artery was not involved in a previous surgery. If both internal iliac arteries have been used for transplantation or the opposite side one is involved by severe stenosing atheromatous plaques, claudication is inevitable as is impotence [30]. However, despite Matsushita’s paper looks very similar to ours, we found it less invasive using a cryopreserved arterial graft since it may allow to spare the internal iliac artery in accurately selected cases, by performing an end to side fashion anastomosis between the repaired arterial segment of the graft and the recipient vessels. Both the donor and the recipient need to be on close and long-term follow up.

Literature lacks clear guidelines on the eligibility of potential living renal donors with asymptomatic FMD. Preliminary assessment of the FMD living donor should always rule out any extra-renal involvement. Whenever possible, resection and reconstruction of the affected arterial segment should be taken into consideration as this condition may progress after implantation. For this reason, recipients of a FMD kidney without surgical correction need an appropriate long term follow-up.

## Data Availability

A copy of written consent, data and material of this study are available from Gecos online repository but restrictions apply to the availability of these data, which were used under license for the current publication, and so are not publicly available. Data are however available from the authors upon reasonable request and with permission of Verona University Hospital and with patient consent.

## Abbreviations

FMD: Fibromuscular dysplasia
CTA: Computed tomography angiography
RAS: Renal artery stenosis
